# Association of *MTHFR* C677T Polymorphism With Antipsychotic-Induced Change of Weight and Metabolism Index

**DOI:** 10.3389/fpsyt.2021.673715

**Published:** 2021-05-21

**Authors:** Yi Su, Hao Yan, Liangkun Guo, Tianlan Lu, CAPOC, Dai Zhang, Weihua Yue

**Affiliations:** ^1^Institute of Mental Health, Peking University Sixth Hospital, Beijing, China; ^2^Key Laboratory of Mental Health, Ministry of Health and National Clinical Research Center for Mental Disorders (Peking University), Beijing, China; ^3^Chinese Antipsychotics Pharmacogenomics Consortium; ^4^Peking-Tsinghua Joint Center for Life Sciences, Peking University, Beijing, China; ^5^PKU-IDG/McGovern Institute for Brain Research, Peking University, Beijing, China; ^6^Department of Translational Medicine Core, Chinese Institute for Brain Research, Beijing, China

**Keywords:** metabolic abnormalities, antipsychotic-induced weight gain, polymorphisms, methylenetetrahydrofolate reductase, schizophrenia

## Abstract

Although antipsychotic medication contributed to the improvement of psychotic symptoms and reduced relapse, it induced weight gain and metabolic syndrome during antipsychotic medication treatment, which was seriously concerning. To investigate the association of methylenetetrahydrofolate reductase (*MTHFR*) gene C677T (rs1801133) polymorphism with antipsychotic-induced weight gain and metabolism parameter change, we employed 1,868 patients with schizophrenia in this study and randomly allocated them to seven antipsychotic medication treatment groups. All patients received antipsychotics monotherapy and were followed up for 6 weeks. Height, body weight, and metabolic parameters of the patients were measured at baseline and at 2, 4, and 6 weeks after antipsychotic treatment. We genotyped blood DNA from patients for *MTHFR* C677T polymorphisms and performed quantitative analyses using analysis of variance (ANOVA) and the analysis of covariance (ANCOVA) among three genotype groups.

We found a predominant association between *MTHFR* C677T and body weight mass index (BMI) change after 6-week risperidone treatment. After 6-week treatment of risperidone, the BMI change rate (%) of *MTHFR* C677 carriers was significantly higher than that of *MTHFR* TT genotype carriers [CC (2.81 ± 6.77)%, CT (3.79 ± 5.22)%, TT (1.42 ± 3.53)%, *F* = 4.749, *P* = 0.009]. Some of the abnormal metabolic parameters were found to be associated with the *MTHFR* 677T, including higher levels of low-density lipoprotein and waist circumference. Validation was performed in an independent cohort, consisting of 252 patients with schizophrenia treated with three atypical antipsychotic drugs. Overall, the *MTHFR* C677 was associated with high risk of antipsychotic-induced weight gain and metabolism abnormalities.

## Introduction

Schizophrenia affects nearly 1% of the world population and is among the top 10 global causes of disability (1). Antipsychotic medications are commonly used to control psychotic symptoms. However, antipsychotic medication can induce weight gain and alteration of metabolic parameters, which not only influence treatment adherence but also lead to diabetes, hyperglycemia, hypertension, and cardiac death (2–5). It is important to notice these changes early on to prevent them from progressing. Recent studies implied that the individual propensity that developed weight gain and the change of metabolic parameters during treatment substantially differed due to genetic factors (6–9).

*MTHFR* encoded methylenetetrahydrofolate reductase, which converts folate into the metabolically active form of 5-methyltetratydrofolate. It plays an important role in the metabolism of folate and homocysteine. It has been reported that the polymorphisms of MTHFR were associated with the metabolic syndrome in both the general population (10–14) and antipsychotic-administrated schizophrenia patients (15, 16), which makes the genetic variant of *MTHFR* a potential predictor for antipsychotic-induced metabolic side effects (e.g., weight gain) (15–17). However, these research were not aligned together, and the influence of the variant of *MTHFR*-C677T on antipsychotic-induced weight gain remained unclear with rare related studies (18).

In this study, we examined the impact of *MTHFR* C677T polymorphism on weight changes and metabolic parameters in schizophrenia patients with antipsychotic treatment.

## Materials and Methods

### Study Design and Participants

The cohort was from the Chinese Antipsychotics Pharmacogenomics Consortium (CAPOC) (19), which was established in 2010, the members of which come from five research centers (Peking University Sixth Hospital, West China Hospital of Sichuan University, the Second Xiangya Hospital of Central South University, Beijing Anding Hospital affiliated to Capital Medical University, and Beijing HuiLongGuan Hospital). It is a 6-week multicenter randomized open-label pharmacogenetic trial designed to identify predictors of antipsychotic drug efficacy and adverse reaction in patients with schizophrenia. Patients were randomly assigned to six groups [aripiprazole, olanzapine, quetiapine, risperidone, ziprasidone, or one of the first-generation antipsychotics (haloperidol or perphenazine)]. All study protocols were approved by the institutional ethics review boards at each site, and written informed consent was obtained. The study was approved by the research ethical committees of local hospitals.

Patients included in this study met the following inclusion criteria: (1) diagnosis of schizophrenia determined by SCID [Structured Clinical Interview of the Diagnostic and Statistical Manual of Mental Disorders, fourth edition (DSM-IV)]; (2) Chinese Han descents; (3) age range from 18 to 45 years; and (4) total score of positive and negative symptom scale (PANSS) ≥ 60 (and scored more than four on at least three positive items). Both first-episode patients (FEP) and relapsed patients with schizophrenia were enrolled from the inpatient departments of the psychiatric hospitals affiliated with CAPOC. The exclusion criteria were as follows: (1) patients who are pregnant or lactating, or menopausal women; (2) patients who require long-acting injectable medication to maintain treatment adherence; (3) patients regularly treated with clozapine for treatment over the past month; (4) patients who have serious suicide attempts or have been experiencing severe excitement and agitation; (5) patients who were treated with electroconvulsive therapy during the last month; (6) patients who have abnormal liver or renal function (aspartate aminotransferase ≥ 80 U/L, alanine aminotransferase ≥ 80 U/L, blood urea nitrogen ≥ 9.75 mmol/L, urine creatinine ≥ 21.6 mmol per day); (7) patients who do not have a legal guardian (it was a hospital stipulation that written informed consent was required from the patient's legal guardian); and (8) patients who have QTc prolongation, a history of congenital QTc prolongation, or recent (within the past 6 months) myocardial infarction.

The replication sample enrolled 252 patients who completed 8 weeks of antipsychotic (olanzapine, quetiapine, and risperidone) monotherapy and related clinical assessment.

### Phenotypes and Definitions

Metabolic syndrome (MetS) was defined according to the new International Diabetes Federation (IDF) definition criteria of the metabolic syndrome. According to the new IDF definition, for a person to be defined as having MetS, he or she must have central obesity (for Chinese, the waist circumference of males should be ≥90 cm, while that of females should be ≥80 cm); besides, they should meet at least any two of the following four factors: (1) raised triglyceride (TG) level, TG ≥ 150 mg/dl (1.7 mmol/L); (2) reduced HDL cholesterol, HDL < 40 mg/dl (1.03 mmol/L) in males and <50 (1.29 mmol/L) in females; (3) raised blood pressure: systolic BP, systolic BP (SBP) ≥ 130 mmHg, or diastolic BP (DBP) ≥ 85 mmHg; and (4) raised fasting plasma glucose ≥ 100 mg/dl (5.6 mmol/L).

### Genotyping

In total, 5-ml peripheral blood samples from subjects were collected in tubes containing EDTA as an anticoagulant and stored at 4°C. Genomic DNA was extracted from the samples using a Qiagen QIAamp DNA Mini Kit (Qiagen GmbH, Hilden, Germany) within 1 week and stored at −70°C for later use. Then, the *MTHFR* C677T (rs1801133) was genotyped with the direct DNA sequencing method. The primer sequences were as follows: 5′ AGC CCA GCC ACT CAC TGT TTT 3′ and 5′ CAG CGA ACT CAG CAC TCC A 3′. PCR amplification was performed in a final volume of 25 μl, using 10 mM Tris-HCl (pH 8.3), 50 mM KCl, 1.5 mM MgCl_2_, 200 μM of each dNTP, 0.4 μM of each primer, 1 U of Taq DNA polymerase, and 30–50 ng of genomic DNA. In the following protocol, DNA was denatured at 94°C for 5 min, amplified by 35-s cycles at 94°C for 30 s and cooled at 64°C for 30 s, 72°C for 1 min, and a final elongation at 72°C for 10 min. For SNP rs1801133, the PCR products were sequenced by DNA sequencing after cleaning the PCR product using BigDye Terminator Cycle Sequencing Ready Reaction Kit with Ampli-Taq DNA polymerase (PE Biosystem). The inner primers were used for the cycle-sequencing reaction, and fragments were separated by electrophoresis on an ABI PRISM 377-96 DNA sequencer (Applied Biosystem). All genotyping was done blind to the knowledge of the subjects' clinical data.

### Statistical Analysis

All of the statistical analyses were carried out using the SPSS 26.0 software (SPSS Inc., Chicago, IL, USA). Before the analysis, we converted the patient's antipsychotic dose to the equivalent dose of chlorpromazine (20, 21). The change of BMI and metabolism parameters was assessed with quantitative analyses. Quantitative analyses were calculated using analysis of variance (ANOVA) among three genotype groups. Furthermore, considering that gender, age, and the dose of antipsychotics may influence weight gain and metabolism parameters, we performed the analysis of covariance (ANCOVA) with sex, age, and drug dose as covariates among three genotype groups.

Metabolic parameters and BMI change were assessed and further explored in FEP patients. Statistical significance was calculated using repeated measures ANOVA to evaluate the evolution of metabolic parameters over the study period in relation to *MTHFR* genotype, and we set sex, age, and drug dose as covariates.

## Results

### Demographic and Metabolic Characteristics of Study Population

A total of 3,030 subjects consented to the study, 279 of them did not meet inclusion criteria, 109 of them dropped out before the second week, 498 of them failed to perform the laboratory test, 47 of them have current treatment with medications known to affect metabolism (e.g., glucocorticoids), 101 of them did not have good-quality DNA available, and 128 of them failed quality control (sex inconsistency, cryptic relatedness). There were 1,868 patients who actually completed 6 weeks of medication, including 947 males and 921 females, with an average age of 30.79 ± 7.89 years old, and there were 252 people in the validation sample (Table 1). We performed ANOVA to test the association of *MTHFR* gene C677T polymorphism with metabolic index at both baseline and after 6-week treatment (Table 2).

**Table 1:** Demographic and clinical characteristics of discovery and validation samples.

	**Discovery**							**Validation**		
	**ARI**	**OLA**	**PERPH**	**HAL**	**QUE**	**RIS**	**ZIP**	**OLA**	**QUE**	**RIS**
	**(*n* = 302)**	**(*n* = 328)**	**(*n* = 158)**	**(*n* = 307)**	**(*n* = 144)**	**(*n* = 330)**	**(*n* = 299)**	**(*n* = 76)**	**(*n* = 55)**	**(*n* = 121)**
Age	31.44 ± 7.57	30.52 ± 7.86	31.96 ± 7.91	33.13 ± 7.59	30.91 ± 7.67	30.86 ± 7.91	30.70 ± 7.86	29.15 ± 8.10	27.11 ± 7.83	29.95 ± 7.93
**Gender**										
Male	150(49.7%)	165(50.3%)	75(47.5%)	67(46.5%)	144(46.9%)	194(58.8%)	152(50.8%)	29 (38.2%)	31(56.4%)	47 (38.8%)
Female	152(50.3%)	163(49.7%)	83(52.5%)	77(53.5%)	163(53.1%)	136(41.2%)	147(49.2%)	47 (61.8%)	24 (43.6%)	74 (61.2%)
First episode	83 (27.5%)	85 (25.9%)	37 (23.4%)	32(22.2%)	78(25.4%)	95(28.8%)	81(27.1%)	14 (18.4%)	21 (38.2%)	52 (43.0%)

*ARI, aripiprazole; OLA, olanzapine; PERPH, perphenazine; HAL, haloperidol; QUE, quetiapine; RIS, risperidone; ZIP, ziprasidone*.

**Table 2:** Association of *MTHFR* C677T polymorphism with antipsychotic-induced metabolic index at both baseline and after 6-week treatment in schizophrenia patients.

	**CC**	**CT**	**TT**	***F***	***P^**†**^***
Sex (male/female)	256/235	449/426	242/260	0.893	0.410
Age (years)	31.04 ± 7.88	30.91 ± 7.63	31.64 ± 7.98	1.428	0.240
**Serum HDL (mmol/L)**					
Baseline	1.31 ± 0.32	1.29 ± 0.33	1.27 ± 0.31	1.957	0.142
End of 6 weeks	1.31 ± 0.31	1.29 ± 0.33	1.26 ± 0.33	2.350	0.096
**Serum LDL (mmol/L)**					
Baseline	2.25 ± 0.72	2.28 ± 0.74	2.38 ± 0.74	3.738	0.024^*^
End of 6 weeks	2.33 ± 0.72	2.39 ± 0.74	2.47 ± 0.79	3.353	0.035^*^
**Serum TG (mmol/L)**					
Baseline	1.06 ± 0.45	1.07 ± 0.48	1.10 ± 0.49	0.759	0.469
End of 6 weeks	1.42 ± 0.66	1.43 ± 0.68	1.45 ± 0.67	0.349	0.706
**Free Serum GLU (mmol/L)**					
Baseline	4.80 ± 0.67	4.80 ± 0.66	4.73 ± 0.64	1.541	0.214
End of 6 weeks	4.70 ± 0.61	4.75 ± 0.60	4.74 ± 0.59	1.120	0.327
**WC (cm)**					
Baseline	78.97 ± 9.56	79.36 ± 9.33	80.28 ± 9.89	2.443	0.087
End of 6 weeks	80.38 ± 9.27	80.86 ± 9.42	81.56 ± 9.89	1.856	0.157
**BMI (kg/m**^**2**^**)**					
Baseline	21.87 ± 3.31	21.98 ± 3.21	22.45 ± 3.52	4.439	0.012^*^
End of 6 weeks	22.33 ± 3.21	22.50 ± 3.19	22.87 ± 3.37	3.542	0.029^*^

*HDL, high-density lipoprotein; LDL, low-density lipoprotein; TG, triglyceride; GLU, glucose; WC, waist circumference; BMI, body mass index*.

*The levels of obesity and metabolic indices in the rs1801133 genotypes are shown as mean ± SD*.

† *One-way ANOVA was performed between rs1801133 and metabolic indices at baseline and after 6-week treatment separately*.

* *P < 0.05*.

### Association Analysis of *MTHFR* Gene C677T Polymorphism With Antipsychotic-Induced Weight Gain

**Figure 1:**
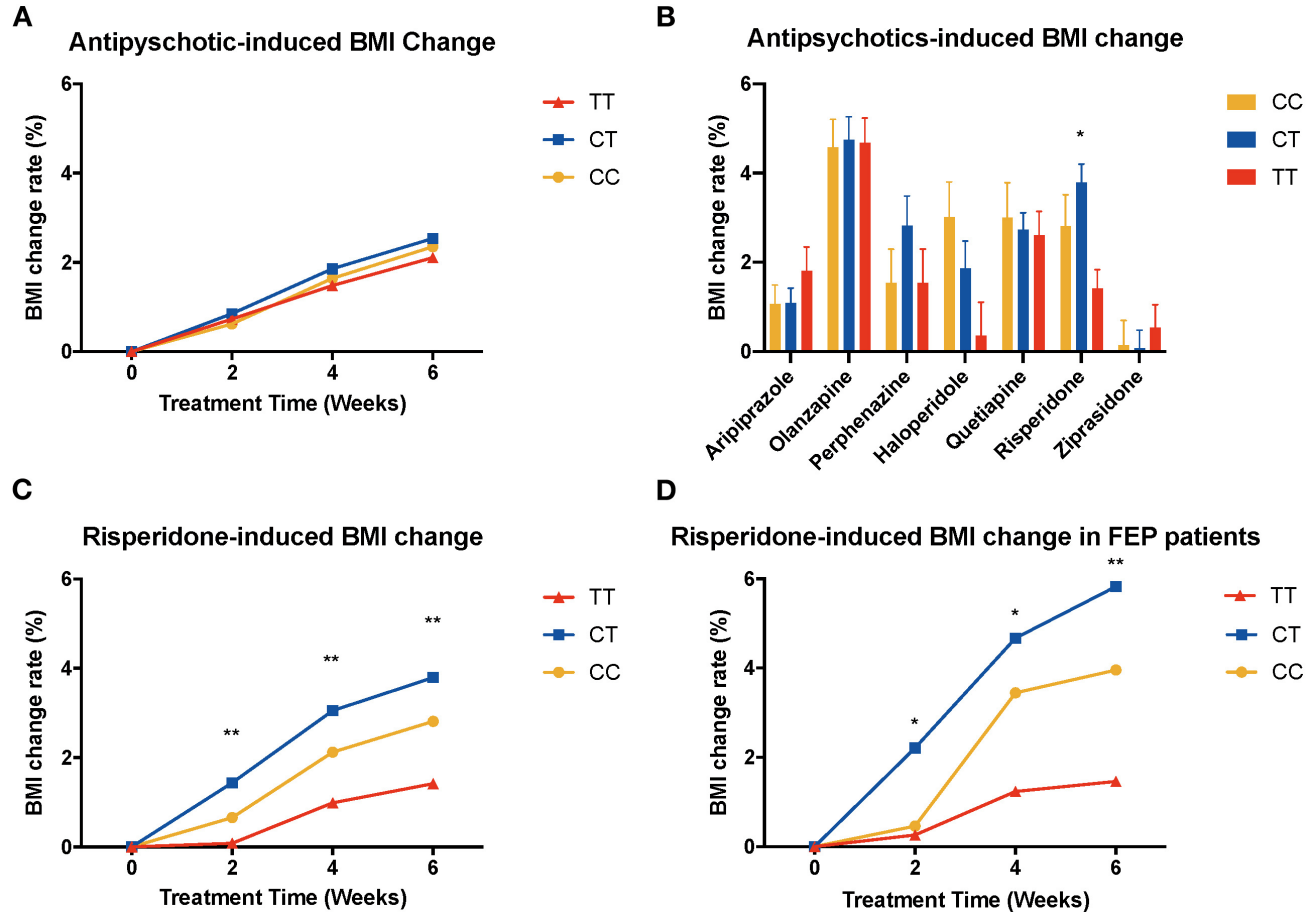
Association of *MTHFR* C677T polymorphism with antipsychotic-induced weight gain in schizophrenia. BMI, body mass index; FEP patients, first-episode patients. **(A)** shows all the patients of different genotypes with increasing tendency; there is no significant difference between these three groups. **(B)** shows different kinds of drugs that induced BMI change rate after 6-week treatment, and in risperidone-treated group, these three genotypes showed significant different change rate (*F* = 4.749, *P* = 0.009). **(C)** shows BMI change rate of risperidone-treated people at different time points (2 weeks, *F* = 5.488, *p* = 0.005; 4 weeks, *F* = 5.981, *P* = 0.003; 6 weeks, *F* = 4.749, *P* = 0.009). **(D)** shows the same pattern in first-episode people (2 weeks, *F* = 3.636, *P* = 0.030; 4 weeks, *F* = 4.066, *P* = 0.021; 6 weeks, *F* = 4.157, *P* = 0.019).

We performed ANOVA to test the association of *MTHFR* gene C677T polymorphism with antipsychotic-induced weight gain (Table 2). Because of the significant difference in baseline BMI (kg/m^2^) before risperidone treatment among the three genotype carriers of *MTHFR* gene C677T polymorphism, we use percentage change of BMI value to correct for baseline BMI. After 6-week treatment, the percentage change in BMI values of CC or CT genotype carriers was higher than that of TT genotype carriers, but there were no significant differences in the whole sample (Figure 1A).

Next, we tested the association with the percentage change in BMI values of patients with specific antipsychotic drug treatment (Figure 1B, Supplementary Figure 1). There was a significant difference between genotypes of *MTHFR* among risperidone-treated patients [6 weeks after treatment, percentage change in BMI value of CC (2.81 ± 6.77), CT (3.79 ± 5.22), TT (1.42 ± 3.53), *F* = 4.749, *P* = 0.009] (Figure 1C).

Previous literature reported that patients who were treated with antipsychotics for the first time (FEP patients) usually present significant weight gain. Therefore, we performed ANCOVA among FEP patients, and we set sex, age, and drug dose as covariates (Supplementary Figure 2). We observed the elevated BMI changing rate in FEP rather than in the relapse group, especially among patients treated with olanzapine (*P* = 0.002), risperidone (*P* = 0.021), ziprasidone (*P* = 0.043), and quetiapine (*P* = 0.002). Besides, we found that FEP patients treated with haloperidol and risperidone showed a significant difference in BMI change among different genotypes of C677T: risperidone-treated group [6 weeks after treatment, percentage change in BMI value of CC (3.96 ± 6.73), CT (5.83 ± 5.40), TT (1.46 ± 3.76), *F* = 4.157, *P* = 0.019] (Figure 1D) and haloperidol-treated group [6 weeks after treatment, percentage change in BMI value of CC (6.27 ± 4.41), CT (1.62 ± 2.66), TT (2.39 ± 3.64), *F* = 4.651, *P* = 0.020].

In the validation sample, we found the same trend of BMI change both in the whole sample and in the risperidone group; the percentage change in BMI values of CC or CT genotype carriers was higher than that of TT genotype carriers, although the result did not reach significant levels (**Figures 3A,B**, Supplementary Figure 8).

### Exploratory Analysis of Metabolic Parameters and BMI

**Figure 2:**
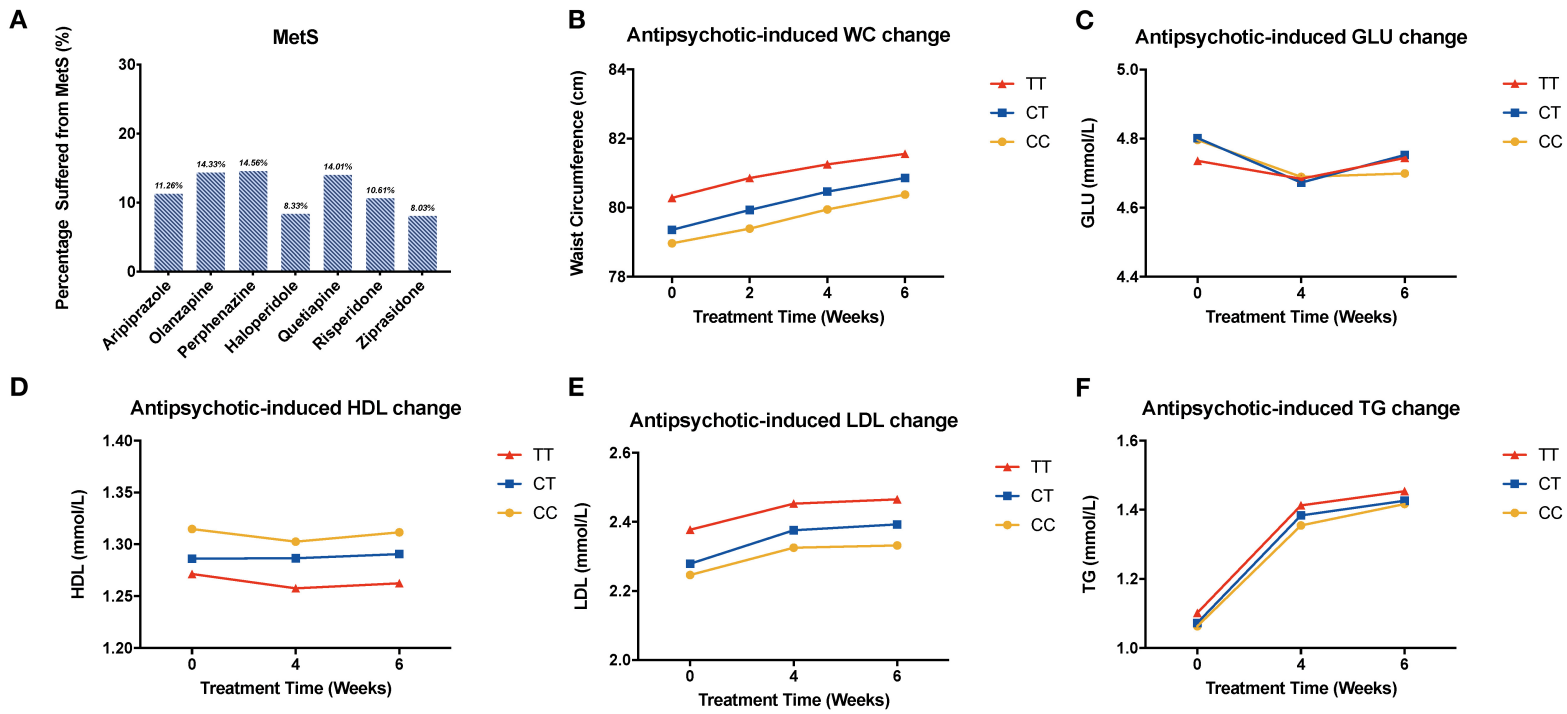
Association of *MTHFR* C677T polymorphism with antipsychotic-induced MetS and metabolic indices change. WC, waist circumference; GLU, glucose; HDL, high-density lipoprotein; LDL, low-density lipoprotein; TG, triglyceride. **(A)** shows the percentage of patients suffering from metabolic syndrome after being treated with different kinds of drugs. **(B–F)** show the change of metabolic components of all the patients.

**Figure 3:**
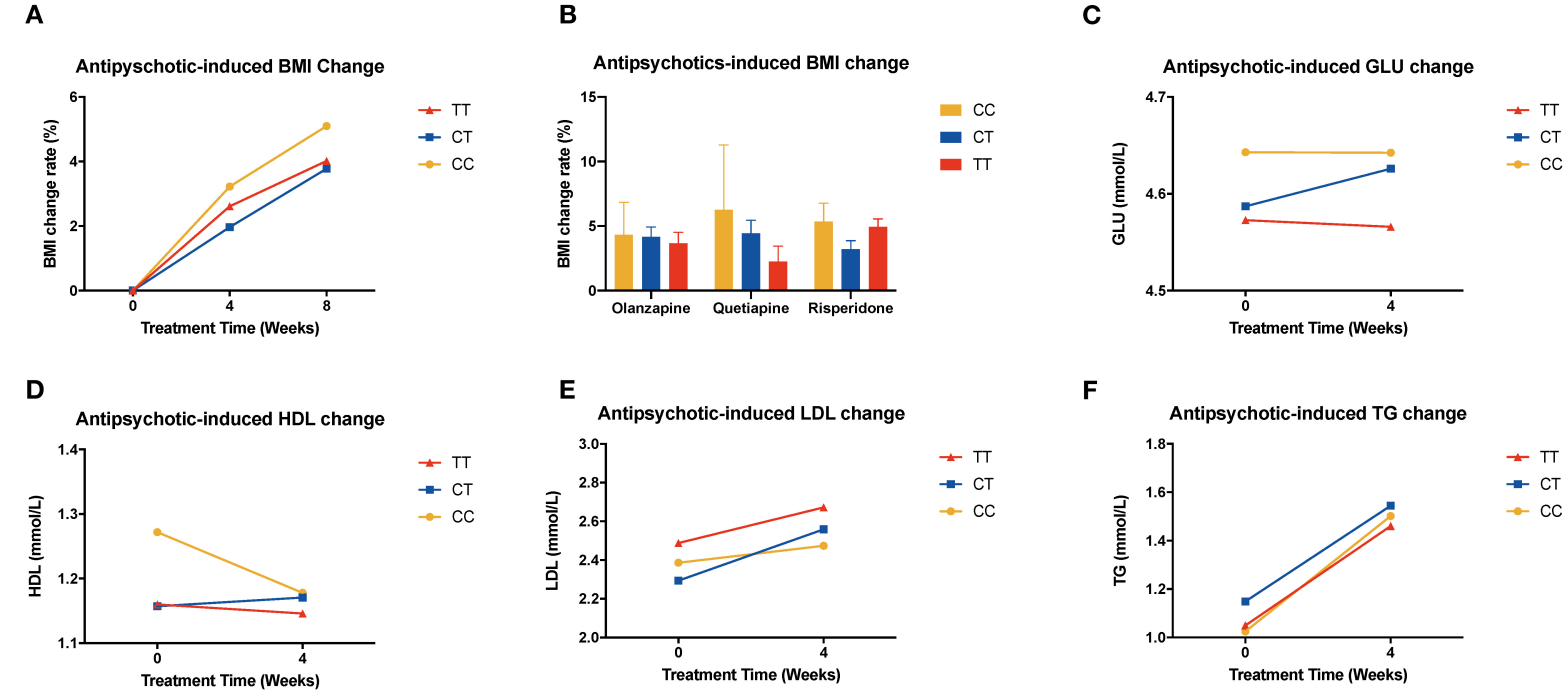
Association of *MTHFR* C677T polymorphism with antipsychotic-induced BMI and metabolic index change in validation sample. **(A)** shows all the patients of different genotypes with increasing tendency; there is no significant difference between these three groups. **(B)** shows different kinds of drugs that induced BMI change rate after 8 weeks of treatment. **(C–F)** show the change of metabolic components of the replication samples.

We compared metabolic indices at different time points and found that the LDL was elevated in antipsychotic medication groups except for ziprasidone. The level increased mostly in the quetiapine-treated group. Besides, all of the patients showed increased level of serum TG and waist circumference, while there is no significant difference among different genotypes of rs1801133 (Figure 2, Supplementary Figures 4 and 6). In addition, the same pattern was shown in the validation sample, and the level of HDL decreased the most in the CC group (Figure 3).

We used repeated measures ANOVA to further explore the relationship of *MTHFR* and metabolic parameters and BMI of antipsychotics-treated subjects, and set sex, age, and drug dose as covariates. There is a significant association between the C677T polymorphism and HDL, LDL, waist circumference, and BMI (Table 3). Among patients treated with haloperidol, the C677T variant showed significant association with HDL (*F* = 4.256, *P* = 0.017); in patients treated with aripiprazole, the variant showed significant association with LDL (*F* = 3.528, *P* = 0.036); in patients treated with risperidone, the variant showed significant association with waist circumference (*F* = 4.038, *P* = 0.019) and BMI (*F* = 3.561, *P* = 0.030). In the validation sample, the patients showed the same tendency but were limited by the sample size; it did not achieve the level of significance (Supplementary Figures 9–12).

**Table 3:** Association of *MTHFR* C677T polymorphism with antipsychotic-induced metabolic parameter change in patients with schizophrenia.

**Characteristic**	***N***	***F***	***P*-value^**†**^**
HDL (mmol/L)	1,544	3.181	0.042^*^
HDL—Haloperidol	118	4.256	0.017^*^
LDL (mmol/L)	1,571	3.338	0.036^*^
LDL—Aripiprazole	259	3.528	0.031^*^
Waist circumference (cm)	1845	4.893	0.008^**^
Waist circumference—Risperidone	333	4.038	0.019^*^
BMI (kg/m^2^)	1,821	3.154	0.043^*^
BMI—Risperidone	322	3.561	0.030^*^

*HDL, high-density lipoprotein; LDL, low-density lipoprotein; WC, waist circumference; BMI, body mass index*.

† *Repeated measures ANOVAs were conducted between rs1801133 and metabolic components. Age, gender, and drug dose were set as covariates*.

* *P < 0.05;*

** *P < 0.01*.

We noticed that gender and time showed an interaction effect, so we performed the subgroup analysis of HDL among males and females, respectively. In males, T-carrier patients treated with olanzapine, perphenazine, quetiapine, and ziprasidone showed a decreasing trend of HDL; meanwhile, in the female group treated with perphenazine and ziprasidone, the trend of HDL was the opposite to that of the male group (Supplementary Figure 7). Other results of this analysis have been listed in the supplementary materials (Supplementary Tables 1–6). In addition, we analyzed the status of patients treated with different drugs after 6-week treatment, and the percentage of patients that suffered from MetS is shown in Figure 2.

## Discussion

In this study, we investigated the association between C677T variant (rs1801133) of *MTHFR* gene, BMI change, and metabolic parameters. The sample size employed and the kinds of antipsychotic medication used in our study went beyond related published research, and we further validated the results in the independent sample. The *MTHFR* protein is prominently involved in the cycles of folate and methionine, which is imperative to the conversion of homocysteine and methionine: *MTHFR* generated 5-methyltetrahydrofolate to initiate the methionine cycle; the end product of *MTHFR*, which is S-adenosylmethionine, can allosterically inhibit this cycle (22). Both of them are crucial for biosynthesis of lipids, nucleotides, and proteins.

A previous study analyzed the association between *MTHFR* 677C/T polymorphism and antipsychotic-induced weight gain and metabolic disturbances: the C677T polymorphism was associated with the alteration of weight and cholesterol level in FEP patients (18). In our study, the *MTHFR* C677 allele carriers showed potent propensity of weight gain than TT homozygous carriers during the acute antipsychotic treatment. We found that this effect was significant in C carriers treated with risperidone and in FEP patients treated with risperidone and haloperidol, but not in other antipsychotic treatments. Besides, we observed the evaluated BMI changing rate in FEP patients but not in relapse patients who are treated with olanzapine. In addition, the order of BMI change rate induced by different drugs is consistent with previous studies (23), and this result drove us to replicate our result in atypical antipsychotic-treated patients.

Prior studies have also shown that the *MTHFR* C677T variant is associated with an increased risk for cardiovascular disease, which is attributed to the elevation in plasma total homocysteine and vascular metabolism of folates (24–26). In our study, we investigated the association of this variant with the metabolic index change induced by antipsychotic treatment, and we found that the *MTHFR* C allele carriers were more likely to increase BMI among risperidone-treated patients as well as the LDL level of haloperidol-treated patients, which are both risk effects of metabolic syndrome.

Because the sample size of each antipsychotic medication is relatively small and the record of lifestyle data was lacking, these preliminary results in specific drugs should be interpreted with caution. Further studies should focus more on prevalence of the metabolic parameters in drug-naive patients and keep a detailed record of the lifestyle data so that we can control the covariates better and find more reliable results. Nevertheless, the repeated measurements of parameters and the larger cohort of patients used in this study provided novel evidence of the impact of *MTHFR* C677 polymorphisms on antipsychotic-induced metabolic syndromes, which contributes to the understanding of antipsychotic side effects and decision of antipsychotic treatment in the clinic.

## Conclusion

Our findings further validated that antipsychotics treatment decreased the level of HDL and increased the levels of TG and LDL, which suggested the high risk of developing metabolic syndrome and increasing the possibility of cardiovascular disease. Besides, we found that people treated with olanzapine, quetiapine, and perphenazine are more likely to suffer from metabolic syndrome.

In addition, the *MTHFR* C allele carriers were more likely to show a tendency to increase BMI among risperidone-treated patients and the LDL level of aripiprazole-treated patients, while the other metabolic indices show the trend but did not reach significant levels.

## Data Availability Statement

The data analyzed in this study is subject to the following licenses/restrictions: The data that support the findings of this study are available from the corresponding author upon reasonable request. Requests to access these datasets should be directed to Weihua Yue, dryue@bjmu.edu.cn.

## Ethics Statement

The studies involving human participants were reviewed and approved by the research ethical committees of local hospitals. The patients/participants provided their written informed consent to participate in this study.

## Author Contributions

All authors have made substantial contributions to all of the following: the conception and design of the study, acquisition of data, analysis and interpretation of data, drafting the article, or revising it critically for important intellectual content.

## Conflict of Interest

The authors declare that the research was conducted in the absence of any commercial or financial relationships that could be construed as a potential conflict of interest.
