# Supplementary material for: Association of MTHFR C677T Polymorphism With Antipsychotic-Induced Change of Weight and Metabolism Index
Source: Front Psychiatry. 2021 May 21;12:673715. doi: 10.3389/fpsyt.2021.673715 (PMC8177429; doi:10.3389/fpsyt.2021.673715)
Supplement: Supplementary file 1 [file Data_Sheet_1.docx]

**Supplementary materials**

**Tables**

Table S1. We performed the repeated ANOVA to analysis the association of *MTHFR* C677T with the HDL change during 6-weeks treatment, and we set sex and age as covariates.

| Drug_HDL | N | *F*-Value | *p*-Value |
| --- | --- | --- | --- |
| All (mmol/L) | 1544 | 3.634 | 0.027* |
| Ariprazole (mmol/L) | 248 | 0.271 | 0.763 |
| Olanzapine (mmol/L) | 279 | 1.303 | 0.273 |
| Perphenazine (mmol/L) | 126 | 0.238 | 0.788 |
| Haloperidole (mmol/L) | 118 | 4.285 | 0.016* |
| Quetiapine (mmol/L) | 256 | 1.708 | 0.183 |
| Risperidone (mmol/L) | 272 | 1.7414 | 0.245 |
| Ziprasidone (mmol/L) | 245 | 0.930 | 0.396 |

Table S2. We performed the repeated ANOVA to analysis the association of *MTHFR* C677T with the LDL change during 6-weeks treatment, and we set sex and age as covariates.

| Drug_LDL | N | *F*-Value | *p*-Value |
| --- | --- | --- | --- |
| All (mmol/L) | 1571 | 4.035 | 0.018* |
| Ariprazole (mmol/L) | 259 | 3.279 | 0.039* |
| Olanzapine (mmol/L) | 281 | 1.098 | 0.335 |
| Perphenazine (mmol/L) | 128 | 0.772 | 0.464 |
| Haloperidole (mmol/L) | 124 | 0.054 | 0.948 |
| Quetiapine (mmol/L) | 258 | 0.429 | 0.651 |
| Risperidone (mmol/L) | 276 | 0.514 | 0.599 |
| Ziprasidone (mmol/L) | 245 | 0.059 | 0.943 |

Table S3. We performed the repeated ANOVA to analysis the association of *MTHFR* C677T with the TG change during 6-weeks treatment, and we set sex and age as covariates.

| Drug_TG | N | *F*-Value | *p*-Value |
| --- | --- | --- | --- |
| All (mmol/L) | 1777 | 0.659 | 0.518 |
| Ariprazole (mmol/L) | 247 | 0.704 | 0.496 |
| Olanzapine (mmol/L) | 275 | 1.336 | 0.265 |
| Perphenazine (mmol/L) | 121 | 0.046 | 0.955 |
| Haloperidole (mmol/L) | 126 | 0.324 | 0.724 |
| Quetiapine (mmol/L) | 240 | 0.983 | 0.376 |
| Risperidone (mmol/L) | 281 | 0.199 | 0.820 |
| Ziprasidone (mmol/L) | 256 | 0.628 | 0.535 |

Table S4. We performed the repeated ANOVA to analysis the association of *MTHFR* C677T with the GLU change during 6-weeks treatment, and we set sex and age as covariates.

| Drug_GLU | N | *F*-Value | *p*-Value |
| --- | --- | --- | --- |
| All (mmol/L) | 1613 | 0.541 | 0.582 |
| Ariprazole (mmol/L) | 262 | 2.330 | 0.099 |
| Olanzapine (mmol/L) | 291 | 0.540 | 0.584 |
| Perphenazine (mmol/L) | 132 | 0.507 | 0.603 |
| Haloperidole (mmol/L) | 121 | 1.455 | 0.238 |
| Quetiapine (mmol/L) | 272 | 0.167 | 0.846 |
| Risperidone (mmol/L) | 280 | 1.583 | 0.207 |
| Ziprasidone (mmol/L) | 255 | 0.919 | 0.400 |

Table S5. We performed the repeated ANOVA to analysis the association of *MTHFR* C677T with the waist circumference change during 6-weeks treatment, and we set sex and age as covariates.

| Drug_ Waist Circumference | N | *F*-Value | *p*-Value |
| --- | --- | --- | --- |
| All (cm) | 1845 | 5.871 | 0.003** |
| Ariprazole (cm) | 309 | 2.853 | 0.059 |
| Olanzapine (cm) | 337 | 0.596 | 0.552 |
| Perphenazine (cm) | 160 | 2.205 | 0.114 |
| Haloperidole (cm) | 142 | 2.092 | 0.127 |
| Quetiapine (cm) | 317 | 0.285 | 0.752 |
| Risperidone (cm) | 333 | 4.027 | 0.019* |
| Ziprasidone (cm) | 299 | 0.476 | 0.622 |

Table S6. We performed the repeated ANOVA to analysis the association of *MTHFR* C677T with the BMI change during 6-weeks treatment, and we set sex and age as covariates.

| Drug_BMI | N | *F*-Value | *p*-Value |
| --- | --- | --- | --- |
| All (mmol/L) | 1821 | 3.747 | 0.024* |
| Ariprazole (mmol/L) | 293 | 2.476 | 0.086 |
| Olanzapine (mmol/L) | 321 | 0.894 | 0.410 |
| Perphenazine (mmol/L) | 154 | 2.168 | 0.118 |
| Haloperidole (mmol/L) | 138 | 1.765 | 0.175 |
| Quetiapine (mmol/L) | 300 | 1.481 | 0.229 |
| Risperidone (mmol/L) | 322 | 3.574 | 0.029* |
| Ziprasidone (mmol/L) | 293 | 0.010 | 0.990 |

**Figures**

**Figure S1.** **BMI change rate induced by antipsychotics treatment.**

C-I reflected different kind of drug group. In group haloperidole, there is significant difference after 4-week treatment in different genotypes (F=3.768, *p*=0.026).

**Figure S2.** **BMI change rate induced by antipsychotics treatment in FEP patients.**

C-I reflected different kind of drug group. In group haloperidole, there is significant difference after 6-week treatment (F=5.680, *p*=0.009).

**Figure S3.** **LDL level change in schizophrenia patients.**

B-H reflected different kind of drug group. In group aripiprazole, there is significant difference before the antipsychotic treatment. baseline (F=3.534, *p*=0.031), 2-week (F=3.869, *p*=0.022).

**Figure S4. TG level change in schizophrenia patients.**

B-H reflected different kind of drug group.

**Figure S5. GLU level change in schizophrenia patients.**

B-H reflected different kind of drug group. In group perphenazine, there is significant difference before the antipsychotic treatment, at baseline (*F*=4.279, *p*=0.016).

**Figure S6. Waist circumference** **change in schizophrenia patients.**

The analysis was performed in males and females respectively. B-H reflected different kind of drug group.

**Figure S7.HDL_sum change in schizophrenia patients**

The analysis was performed in males and females respectively. B-H reflected different kind of drug group.

**Figure S8. BMI change rate induced by antipsychotics treatment (replication).**

A-C reflected the BMI change in different kind of drug group of replication sample.

**Figure S9. LDL level change in schizophrenia patients (replication).**

A-C reflected the LDL change in different kind of drug group of replication sample.

**Figure S10. TG level change in schizophrenia patients (replication).**

A-C reflected the TG change in different kind of drug group of replication sample.

**Figure S11. GLU level change in schizophrenia patients. (replication).**

A-C reflected the GLU change in different kind of drug group of replication sample.

**Figure S12. HDL level change in schizophrenia patients. (replication).**

A-C reflected the HDL change in different kind of drug group of replication sample.
